# Aesthetics outcome of immediately restored single implants placed in extraction sockets in the anterior maxilla: A case series study

**DOI:** 10.34172/joddd.2020.007

**Published:** 2020

**Authors:** Amin Saedi Germi, Vadoud Ghasemi Barghi, Karim Jafari, Rahman Nemati, Saeed Yeganzad

**Affiliations:** ^1^Student Research Committee, Faculty of Dentistry, Ardabil University of Medical Sciences, Ardabil, Iran; ^2^Department of Periodontics, Faculty of Dentistry, Ardabil University of Medical Sciences, Ardabil, Iran; ^3^Department of Orthodontics, Faculty of Dentistry, Ardabil University of Medical Sciences, Ardabil, Iran

**Keywords:** Aesthetics, immediate loading, single-tooth implant, soft tissue

## Abstract

***Background.*** Immediate single implant placement and restoration (IIR) is recognized as a novel method and is the main request of many patients. This study was designed to evaluate the aesthetic outcomes of immediately restored single implants placed in extraction sockets in theanterior maxilla.

***Methods.*** In this case series study, 18 patients were selected from two private clinics after placing a single-tooth implant in the anterior maxilla. Immediate provisional crowns were delivered on the following day or at most 48 hours later, and guidelines were provided. The Pink Esthetic Score (PES) questionnaire was used at 6- and 12-month follow-ups to assess aesthetic outcomes. Data were analyzed with single t-test and dependent t-test.

***Results.*** In general, the results showed that the status of the mesial papilla, distal papilla, curve of the facial soft tissue line, level of the facial peri-implant mucosa and root convexity soft tissue in IIR method were optimal (P<0.05), with total PES means of 9.44±0.783 and 8.58±1.003 after 6 and 12 months, respectively. Also, the results showed a significant difference in PES between the 6-month and 12-month intervals (P<0.05).

***Conclusion.*** IIR is a viable method that resulted in optimal aesthetic outcomes based on PES in the short term. Considering its confirmation in this study and previous studies, it is recommended that dentists apply this method.

## Introduction

Nowadays, aesthetics outcome is an important factor in all the dental procedures. In the early years of implant therapy, the momentous issue was osseointegration, and many studies focused on the factors affecting it.^1^ The basic aim was to restore function to edentulous patients; however, over time, with the exponential success of osseointegration, researchers re-directed their attention to the aesthetic outcomes of restorations.^1^

Since the first report of implant placement in a freshly extracted socket,^2^ studies have shown that early/immediate implant placement would yield predictable outcomes for implant treatment.^3,4^ Implant placement in a freshly extracted socket and its immediate loading has many advantages, such as a decrease in the duration of surgery sessions, reduced healing time, psychological and functional advantages of early restoration, and simultaneous soft tissue formation.^5,6^ Moreover, according to some studies, this could be beneficial in reducing unfavorable soft tissue changes during the surgical procedure and promoting the establishment of aesthetics.^7^

Belser et al^7^ showed that all the 45 anterior maxillary single-tooth implants fulfilled the strict success criteria, including the absence of peri-implant radiolucency, implant mobility, suppuration, and pain. They also concluded that the immediate loading of anterior maxillary single-tooth implants was successful in terms of aesthetic aspects.

Immediate single-implant placement and restoration (IIR) are regarded as a newer method compared to other implant surgical methods. Research in this aspect and provision of sufficient evidence for dentists regarding the IIR technique could help dentists to decide whether to use the IIR method or not, considering that immediate implant placement in the anterior maxillary edentulous region is the main request of many patients. Therefore, on-spot treatment of patients might bring great satisfaction. Therefore, this study aimed to assess the aesthetic outcomes of soft tissues in the anterior maxillary single-tooth implants in the IIR method.

## Methods

In this case series study, eight male and ten female patients were selected from two private clinics in Ardabil, Iran, who needed single-tooth implants in the anterior maxilla. The maxillary premolar teeth were regarded as candidates when they were in the aesthetic zone.

Inclusion criteria were patients in need of fresh socket implants with immediate restorations in the anterior maxilla and patients’ consent as well. Exclusion criteria were as follows: poor oral hygiene with plaque index>20%, uncontrolled systemic disease and American Society of Anesthesiologists’ class III and IV patients, acute infection at the implant site along with suppuration, absence of one or two proximal teeth, non-treated chronic periodontitis or aggressive periodontitis, thin scalloped gingival biotype, and dehiscence or fenestration in the anterior maxillary buccal region necessitating guided bone regeneration (GBR) procedure.

### Measurement and evaluation indices

To assess aesthetic status, the Pink Esthetic Score (PES) questionnaire was used. The questionnaire evaluates PES index score.^7^

**Figure 1:**
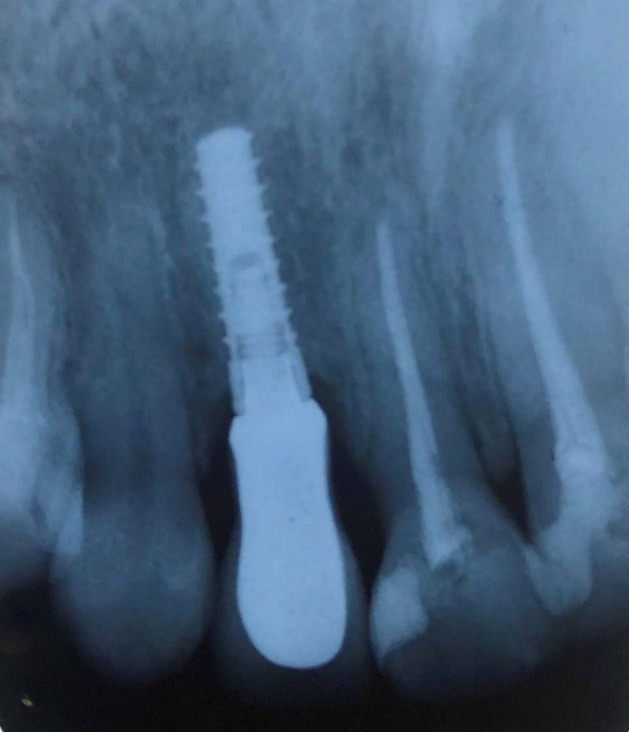
Periapical view of the fresh socket implant at 12-month follow-up recall (a 24-year-old female, right maxillary lateral incisor).

The surgery was carried out under local anesthesia, using a minimally invasive technique. In cases with buccal bone fracture occurring during extraction, the whole implantation process was ceased, and the treatment plan was changed to delayed loading of the prosthesis or a GBR procedure. A periotome was used for cutting supra-crestal fibers, and the extraction of teeth was carried out using minimal buccolingual forces. Simultaneous extraction and implantation were carried out 2 mm apical to the buccal cortex at least and 2 mm lingual to the buccal table. This procedure was implemented by 35 N.cm^2^ torque at least (Figure 1).

Evaluations were carried out for the possibility of establishing proper 3-dimensional prosthetic position, confirmation of overjet and overbite in implant placement, and surgery procedure by the use of paralleling pins. Then the impression taking instruments, including impression copings, analogs attached to the fixtures, and impressions, were sent to the laboratory to prepare provisional crowns, which were delivered to the patients within 24 hours. Finally, the distance between the implant and buccal plate was filled with allograft (produced by the Hamanandsaz Baft Kish Company) to minimize the possible risk of hard and soft tissue collapse, sutured if required, and the implant site was covered by healing abutment until installation of provisional crown was done (within 24 hours). Amoxicillin, 500‒750 mg three times a day for seven days, Ibuprofen, 200 mg three times a day for three days and chlorhexidine mouthwash twice a day for 13 days were prescribed. Each patient returned on the following day, or at most 48 hours later, the immediate provisional crown was delivered, and guidelines were provided.

**Figure 2:**
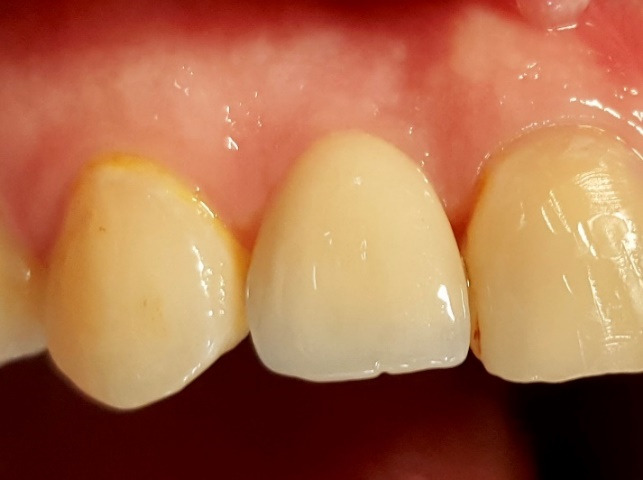
Fixed restoration and gingival soft tissue evaluation at 12-month follow-up recall (the same patient).

The patients were recalled for the evaluation of treatment results after two weeks, and provisional restorations were replaced with fixed restorations afterward. The patient’s PES parameters were measured by the same examiner in 6-month and 12-month follow-up recalls (Figure 2), with the 6-month interval before fixed restoration delivery and the 6-month interval after fixed restoration delivery.

### Statistical analysis

Data were reported using means, medians, and standard deviations (SD). Analyses, including calculation of descriptive and analytical statistics presented in Excel (Microsoft, Redmond, WA, USA), were performed using SPSS 22, with the parametric test of single t-test and dependent t-test to compare PES index with the threshold of clinical acceptability set at 6.^7^

## Results

### Evaluation of soft tissue aesthetic indices 6months after IIR in the anterior maxilla

According to Table 1, in the mesial and distal papilla indices, 88.8% and 77.7%, in the curve of the facial soft tissue line index, 100%, in the level of the facial peri-implant mucosa index, 83%, in the root convexity soft tissue, 89%, and in the total PES, 62% of implants gained a complete score. Also, the central incisor gained complete scores for soft tissue aesthetic indices six months after IIR. Table 2 presents the summary of PES scores, including means and SDs.

**Table 1:** Detailed PES of all the 18 implants after 6 and 12 months

**Patient**	**Implant site**	**PES after 6 months**						**PES after 12 months**					
		**Mesial papilla**	**Distal papilla**	**Curvature of the facial soft tissue line**	**Level of the facial peri-implant mucosa**	**Root convexity soft tissue**	**Total PES**	**Mesial Papilla**	**Distal Papilla**	**Curvature of the facial soft tissue line**	**Level of the Facial peri-implant mucosa**	**Root convexity soft tissue**	**Total PES**
**1**	7	2	2	2	2	2	10	2	2	2	2	1	9
**2**	6	2	2	2	2	2	10	1	2	2	2	2	9
**3**	10	1	1	2	2	2	8	1	1	2	2	2	9
**4**	8	2	2	2	2	2	10	2	2	2	2	2	10
**5**	8	2	2	2	2	2	10	2	2	2	2	2	10
**6**	11	2	1	2	2	2	9	2	1	2	2	1	9
**7**	8	1	2	2	2	2	10	1	2	2	2	1	9
**8**	9	2	2	2	2	2	10	2	2	2	2	2	10
**9**	6	2	2	2	2	2	10	2	2	2	1	1	8
**10**	8	2	2	2	2	2	10	1	2	2	2	1	8
**11**	10	2	2	2	2	2	10	2	2	1	1	2	7
**12**	9	2	2	2	2	2	10	2	2	2	2	1	9
**13**	12	2	2	2	1	2	9	2	1	1	2	1	7
**14**	7	2	1	2	1	2	8	2	1	2	2	2	8
**15**	12	2	2	2	2	1	9	2	1	2	2	2	9
**16**	9	2	2	2	1	1	8	2	2	2	1	1	8
**17**	9	2	1	2	2	2	9	1	1	2	1	1	6
**18**	12	2	2	2	2	2	10	2	2	1	1	1	7

**Table 2:** Summarized PES of the 18 implants after 6 and 12 months

**PES after 6 months**						
	**Mesial papilla**	**Distal papilla**	**Curvature of facial mucosa**	**Level of facial mucosa**	**Root convexity soft tissue**	**Total PES**
**Maximum**	2	2	2	2	2	10
**Minimum**	1	1	2	1	1	8
**Mean**	1.88	1.77	2	1.83	1.88	9.44
**SD**	0.323	0.427	0	0.383	0.323	0.783
**PES after 12 months**						
	**Mesial papilla**	**Distal papilla**	**Curvature of facial mucosa**	**Level of facial mucosa**	**Root convexity soft tissue**	**Total PES**
**Maximum**	2	2	2	2	2	10
**Minimum**	1	1	1	1	1	6
**Mean**	1.72	1.66	1.83	1.72	1.38	8.58
**SD**	0.46	0.485	0.383	0.46	0.501	1.003

### Evaluation of soft tissue aesthetic indices 12 months after IIR in the anterior maxilla

According to Table 1, in the mesial and distal papilla indices, 72% and 66.6%, in the curve of the facial soft tissue line index, 83%, in the level of the facial peri-implant mucosa index, 72%, in the root convexity soft tissue, 44%, and in total PES, 17% of implants gained a complete score. Also, the central incisor gained complete scores for soft tissue aesthetic indices 12 months after IIR. Table 2 summarizes the PES scores, including means and SDs.

Both the single t-test and dependent t-test results showed significant differences in the soft tissue aesthetics (P<0.05) between the means acquired 6 months and 12 months after IIR (considering a threshold of clinical acceptability ≥6). Single t-test results indicated favorable soft tissue aesthetics 6 and 12 months after IIR in the anterior maxilla. The latter test showed a significant reduction in PES means after 12 months (8.58±1.003) compared to that after six months (9.44±0.783).

The single t-test results showed favorable outcomes in each PES index six and 12 months after IIR in the anterior maxilla. However, dependent t-test results for the whole PES indices (between 6-month and 12-month periods) showed no significant difference except for root convexity soft tissue index (P>0.05).

## Discussion

Establishing aesthetics and meeting the patient’s demand in a minimum period of time is a challenging yet important goal in implant therapy. Considering presurgical soft tissue assessment and accurate surgical protocols, it is possible to reduce the problems regarding aesthetics and decrease the long duration of therapy.^8^

In this case series study, aesthetic outcomes of 18 single-tooth implantations by the IIR method were assessed in 6-month and 12-month follow-up sessions. The results demonstrated that PES index means in the anterior maxillary teeth with the IIR method after 6 and 12 months were 9.44±0.783 and 8.58±1.003, respectively. None of the 18 implants gained a score below 6 (6 has been approved in different studies, as the clinical threshold acceptability of soft tissue).^2,9,10^ The mean acquired in this study for soft tissue status is higher than the clinical acceptability threshold. Similar results were observed in other studies. Belser et al^7^ estimated a total PES mean of 7.8±0.88 out of 10 after a 2‒4-year follow-up, with Hartlev et al^9^ and Mangano et al^10^ reporting 9.9 out of 12 after 33 months of follow-up and 8.1±1.5 out of 10 after 3-4 year follow-up, respectively.

Furthermore, the results of the current study showed a favorable PES mean in the anterior maxillary teeth with the IIR modality. There was a significant difference in the total PES mean in anterior maxillary implants after six and 12 months. Previous studies showed that the alveolar bone remodeling around the implant was a maximum of 1 mm (in immediate provisional crown loading).^11,12^ Ross et al^13^ showed that the highers gingival recession occurred within the first three months, between implant placement/provisionalization and definitive restoration, with the implant diameter, gingival biotype, surgical technique, and the reason for tooth loss influencing the amount of gingival recession. However, these statistical results might be different from those of clinical observations. The results of PES indices after 12 months decreased compared to the 6-month period, but they were acceptable.

Belser et al^7^ and Hartlev et al^9^ showed that loading of immediate single-tooth implants in the anterior maxilla was aesthetically successful and favorable, and they estimated a total PES mean higher than the clinical acceptability threshold. Furthermore, Raes et al^14^ reported a success rate of 98% for loading of the immediate single-tooth implant.

The results of the present study are consistent with those reported by Belser et al^7^ and Hartlev et al,^9^ indicating favorable status of mesial papilla, distal papilla, curve of the facial soft tissue line, level of the facial peri-implant mucosa, and root convexity soft tissue of the ‎anterior maxillary region with the IIR method. According to Belser et al,^7^ the highest score was achieved for the curvature of facial mucosa index.

In this study, approximately 72% and 67% of patients achieved a complete score after a 1-year follow-up in the mesial and distal papilla indices, respectively, while Belser et al^7^ reported 60% and 28% of patients achieved a complete score in the mesial an distal papilla indices in 2‒4-year follow-up and also Hartlev et al^9^ showed 44% and 37% of patients achieved a complete score in the mesial an distal papilla indices in a 33-month follow-up. Subsequently, the size of the dark triangle in the proximal contacts in our study was less than other studies’ results. The reason for the difference in results could related to two issues: first, we used allografts for the buccal gap filling, while in similar study^14^ xenografts or synthetic materials were used and secondly, due to short term following up sessions (difference in soft tissue remodeling duration).

The results of this study and other studies might facilitate decision-making for dentists in using the IIR technique.^7,9,10,13,14^ Immediate implantation of the anterior maxillary region is the most prevailing request of patients. Overall, the results of this new method are favorable in terms of aesthetics and therefore advised. Also, the expertise of the implant surgeon should be considered in using this technique.

## Conclusion

IIR technique is a viable method showing optimum aesthetic results in terms of PES at the short-term follow-up, as confirmed in previous studies. The results showed significant differences in soft tissue aesthetics after 6 and 12 months. By precise patient selection and by observing the IIR method’s approved guidelines, this method can be used favorably. In order to precisely assess the impact of the IIR method on soft tissue indices, it is advisable to increase the number of patients and prolong the follow-up periods in future studies.

## Authors’ Contributions

The concept and design of the study were developed by VG. Data entry and statistical analyses were carried out by AS and SY. KJ and RN were responsible for interpretation of data. VG revised it critically for important intellectual content. AS edited and drafted the manuscript. All authors have read and approved the final manuscript.

## Acknowledgments

None.

## Funding

The study was funded by the Research Council of Ardabil University of Medical Sciences.

## Competing Interests

The authors declare no conflict(s) of interest related to the publication of this work.

## Ethics Approval

This research was approved by Research Ethics Committee of Ardabil University of Medical Sciences.
